# Analysis of the Prevalence of Mild Cognitive Impairment and its Influencing Factors in the Elderly Population in Huzhou City

**DOI:** 10.62641/aep.v53i3.1722

**Published:** 2025-05-05

**Authors:** Weiliang He, Zheli Chen, Liang Xu, Fei Fang, Xin Zu, Xilong Jin, Jing Chen

**Affiliations:** ^1^Department of Elderly Psychiatry, The Third People’s Hospital of Huzhou City, 313000 Huzhou, Zhejiang, China; ^2^Department of Geriatrics, The Third People’s Hospital of Huzhou City, 313000 Huzhou, Zhejiang, China; ^3^Department of Psychiatry, The Third People’s Hospital of Huzhou City, 313000 Huzhou, Zhejiang, China; ^4^Department of Neurology, The Third People’s Hospital of Huzhou City, 313000 Huzhou, Zhejiang, China

**Keywords:** mild cognitive impairment, epidemiology, the elderly population, influencing factor

## Abstract

**Background:**

Mild cognitive impairment (MCI) is a critical stage in the development of Alzheimer’s disease, and early intervention in patients during this stage may reverse or delay their disease progression. As one of the regions with severe aging in China, it is necessary to understand the prevalence of MCI in Huzhou and adopt effective intervention measures. The study was aimed to investigate the prevalence rate and influencing factors of MCI in the elderly population in Huzhou city.

**Methods:**

A cross-sectional study was conducted involving 800 elderly residents of Huzhou city. The Montreal Cognitive Assessment (MoCA) and the activity of daily living (ADL) were used to assess the occurrence of MCI in the elderly. The influencing factors of MCI were investigated by univariate analysis and multi-factor analysis.

**Results:**

A total of 800 questionnaires were sent out in this survey, and 778 were effectively collected, with an effective recovery rate of 97.25%. Among 778 elderly people in Huzhou city, 668 had normal cognitive function, 82 had MCI, and 28 had dementia, the prevalence rate of MCI was 10.54% (82/778). According to the presence or absence of MCI, the patients were divided into an MCI group (*n* = 82) and a non-MCI group (*n* = 668). Female (*p* = 0.026), high age (*p* = 0.009), low Community Screening Instrument for Dementia (CSI-D) score (*p* = 0.007), high Dementia Screening Questionnaire (AD8) score (*p* < 0.001), high Patient Health Questionnaire Depression Scale (PHQ-9) score (*p* = 0.037) were all risk factors for MCI in the urban elderly population of Huzhou City.

**Conclusion:**

The prevalence of MCI in the elderly population in Huzhou City is high, and its occurrence is closely related to many factors. It is necessary to increase attention to the high-risk population of MCI and implement targeted intervention measures to improve their cognitive function and improve the quality of life of the elderly population.

## Introduction

Senile cognitive dysfunction is a prevalent neurological degeneration, second only to cerebrovascular disease, and is a common neurological disorder [1, 2]. Studies have shown that with the intensification of population aging, the prevalence rate of cognitive dysfunction in people over 60 years old in China has been rising year by year, which has brought a heavy burden to society and families [3, 4]. Mild cognitive impairment (MCI), first characterized by Petersen RC *et al*. [5] 2014, describes a specific state of cognitive decline and is considered an intermediate stage between normal aging and dementia. Although MCI patients do not meet the diagnostic criteria for dementia and generally maintain basic daily living skills, they often exhibit declines in cognitive functions such as memory, executive function, language, and attention, as well as difficulties with complex daily living activities [6].

Studies have shown that MCI is a key stage in the development of Alzheimer’s disease [7, 8]. Compared to the annual incidence of dementia in the normal population of 1% to 2%, the number of MCI patients developing dementia every year accounts for 10% to 15% [9]. Alzheimer’s disease, especially in its advanced stages, imposes a significant burden on the families of the patient due to the lack of effective treatments and the need for long-term care, making it a major contributor to national healthcare costs. Early intervention during the MCI stage may delay or even prevent progression to dementia [10]. Therefore, early screening and analysis of MCI-related factors are crucial for developing preventive and therapeutic strategies to improve cognitive function and reduce the socioeconomic burden on families and society.

While large-scale epidemiological surveys have been conducted across China, there is a lack of systematic studies in Zhejiang, especially in Huzhou, a region with a high prevalence of aging. Understanding the prevalence and risk factors of MCI in Huzhou is essential for implementing effective interventions. This study aimed to investigate the prevalence and determinants of MCI in the elderly population of Huzhou and provide scientific foundation for prevention, early detection, and intervention. The findings from this research will contribute to enhancing cognitive function, improving quality of life, and promoting healthy aging in the elderly population.

## Material and Methods

### Study Participants

Eight hundred elderly people aged 60 and above were selected by multi-stage stratified cluster random sampling method from January 2022 to December 2022. The sample size was estimated using the formula: n = (uα/δ)^2^ × p × (1 – p), with α = 0.05, uα = 1.96, and allowable error (δ) of 0.03. Based on an estimated MCI prevalence rate (p) of 0.212, the required sample size was calculated to be 713. Accounting for a 10% inefficiency rate, the sample size was expanded by 10% to 785 cases and rounded to 800 cases. The specific sampling method is as follows: 2 streets are randomly selected from the 7 streets of Aishan, Feiying, Yuehe, Chaoyang, Hudong, Longquan and Huanzhu in Wuxing District of Huzhou, and then 2 communities are randomly selected from these 2 streets, a total of 4 communities. From each community, 2 residential areas were selected, resulting in 8 residential areas where 100 individuals were screened, totaling 800 participants. Out of 800 questionnaires distributed, 778 were completed, yielding an effective response rate of 97.25%.

Inclusion criteria: (1) Age 60 and above; (2) Residence in the community for at least one year; (3) Ability to understand and complete interview. Exclusion criteria: (1) Sensory impairment significantly affecting data collection (e.g., severe deafness, blindness); (2) Presence of mental disorders; (3) Acute phase of major physical illnesses (e.g., stroke).

### Procedures

All raters received consistent training. The purpose, process, significance, and estimated study duration were explained to participants, and informed consent was obtained. Participants were instructed to complete the questionnaire independently. For some patients who cannot fill in the questionnaire due to illness or educational level, the investigator will ask them one by one and record them truthfully. All questionnaires were collected and verified on the same day by designated personnel. The questionnaire included the following: (1) General information: marital status, occupation, educational level, nature of residents, way of living, smoking history (smoking >4 cigarettes/week in the past 6 months), alcohol consumption (≥25 g/day for men, ≥15 g/day for women), medical history (e.g., insomnia, depression, hypertension, diabetes, coronary heart disease, cerebral infarction, cerebral hemorrhage, hyperthyroidism, hypothyroidism, hearing impairment, visual impairment, taste impairment, smell impairment, chronic gastritis, enteritis, hepatobiliary disease, traumatic brain injury), surgical history, family history, family history of dementia, family history of depression, family history of neurasthenia. (2) Cognitive assessments: Scores from the Community Screening Instrument for Dementia (CSI-D) [11] (coefficient alpha = 0.9157 [12]), Dementia Screening Questionnaire (AD8) [13] (coefficient alpha = 0.84 [14]), Patient Health Questionnaire Depression Scale (PHQ-9) [15] (coefficient alpha = 0.894 [16]), and Athens Insomnia Scale (AIS) [17] (coefficient alpha = 0.87 [18]). MCI diagnostic criteria: The patient’s cognitive function was diagnosed according to Petersen’s MCI diagnostic criteria [19]: (1) Memory complaints confirmed by the informant; (2) Memory impairment on the Montreal Cognitive Assessment (MoCA) [20] (coefficient alpha = 0.75 [21]), with 11 points ≤ MoCA score ≤ 15 points for illiterates, 14 points ≤ MoCA score ≤ 20 points for primary school literacy students and 16 points ≤ MoCA score ≤ 23 points for those with junior high school education or above; (3) Normal overall cognitive changes; (4) Normal daily living activities [activity of daily living (ADL) score ≤22 points [22] (coefficient alpha = 0.827 [23])]; (5) Did not meet the diagnostic criteria for dementia.

### Statistical Analysis

Statistical analysis was performed using SPSS v23.0 (IBM SPSS Corp.; Armonk, NY, USA). Categorical variables were analyzed using the Chi-Squared or Fisher exact tests, expressed as frequencies (n). The Shapiro-Wilk normality test assessed the normality of continuous data (age and CSI-D, AD8, PHQ-9, AIS scores). Normally distributed data were presented as mean ± standard deviation ($\bar{x}$ ± *s*) and analyzed using the independent samples *t*-test. Non-normally distributed data were expressed as median (min, max) and analyzed using the Mann-Whitney U test. Univariate analysis and multiple logistic regression analysis were performed to identify factors influencing MCI in the elderly population. A *p*-value < 0.05 was considered statistically significant.

## Results

### Prevalence of MCI 

Among the 778 elderly participants in Huzhou City, 668 had normal cognitive function, 82 had MCI, and 28 had dementia, resulting in an MCI prevalence of 10.54% (82/778).

### Univariate Analysis of MCI

Participants were categorized into an MCI group (n = 82) and a non-MCI group (n = 668) based on cognitive status. Univariate analysis identified gender, age, occupation, educational level, and scores on the CSI-D, AD8, PHQ-9, and AIS scales as factors associated with MCI in the elderly population of Huzhou City (Table 1).

**Table 1.** **Univariate analysis of MCI**.

Variable		n	Non-MCI group (n = 668)	MCI group (n = 82)	*t/χ^2^/Z*	*p*-value
Gender (n)					6.295	0.012
	Male	354	326	28		
	Female	396	342	54		
Age (years)			70.31 ± 6.06	72.99 ± 6.88	3.717	<0.001
Marital status (n)					-	0.136
	Married	656	586	70		
	Unmarried	1	0	1		
	Divorced	8	8	0		
	Widowed	85	74	11		
Occupation (n)					-	0.037
	Retired	520	461	59		
	Retirement	8	7	1		
	Farming	177	155	22		
	Employed	45	45	0		
Education level (n)					11.503	0.042
	No formal education	145	120	25		
	Primary school	243	215	28		
	Junior high school	207	186	21		
	High school/vocational school	110	104	6		
	College/vocational college	19	18	1		
	University or higher	26	25	1		
Nature of resident (n)					0.033	0.856
	Rural	304	270	34		
	Urban	446	398	48		
Living arrangement (n)					-	0.053
	Living alone	160	139	21		
	With children	47	45	2		
	In elderly care	116	100	16		
	With spouse	426	384	42		
	With caregiver	1	0	1		
Smoking status (n)					2.445	0.294
	Never smoked	502	441	61		
	Quit smoking	96	87	9		
	Current smoker	152	140	12		
Drinking status (n)					1.784	0.410
	Never drank	541	480	61		
	Quit drinking	55	47	8		
	Current drinker	154	141	13		
CSI-D score (points)			9 (8, 9)	8 (7, 9)	5.358	<0.001
AD8 score (points)			0 (0, 1)	3 (2, 5)	12.092	<0.001
PHQ-9 score (points)			0 (0, 2)	2 (1, 5)	6.519	<0.001
AIS score (points)			1 (0, 3)	3 (1, 6)	4.666	<0.001
Insomnia (n)					0.033	0.856
	Yes	35	31	4		
	No	715	637	78		
Depression (n)					-	1.000
	Yes	1	1	0		
	No	749	667	82		
Hypertension (n)					0.643	0.423
	Yes	505	453	52		
	No	245	215	30		
Diabetes (n)					0.020	0.887
	Yes	142	126	16		
	No	608	542	66		
Coronary heart disease (n)					0.343	0.558
	Yes	78	71	7		
	No	672	597	75		
Cerebral infarction (n)					1.908	0.167
	Yes	47	39	8		
	No	703	629	74		
Cerebral hemorrhage (n)					0.407	0.523
	Yes	2	1	1		
	No	748	667	81		
Hyperthyroidism (n)					0.407	0.523
	Yes	2	2	0		
	No	748	666	82		
Hypothyroidism (n)					0.104	0.747
	Yes	7	6	1		
	No	743	662	81		
Hearing disorder (n)					0.474	0.491
	Yes	23	22	1		
	No	727	646	81		
Visual disturbance (n)					0.000	1.000
	Yes	41	37	4		
	No	709	631	78		
Chronic gastritis (n)					0.543	0.461
	Yes	66	57	9		
	No	684	611	73		
Enteritis (n)					-	0.381
	Yes	12	12	0		
	No	738	656	82		
Hepatobiliary disease (n)					0.036	0.849
	Yes	60	53	7		
	No	690	615	75		
Cerebral trauma (n)					0.799	0.371
	Yes	7	5	2		
	No	743	663	80		
Surgical history (n)					2.078	0.149
	Yes	429	376	53		
	No	321	292	29		
Family history of dementia (n)					-	0.620
	Yes	11	11	0		
	No	739	657	82		
Family history of depression (n)					-	1.000
	Yes	5	5	0		
	No	745	663	82		
Family history of neurasthenia (n)					-	1.000
	Yes	1	1	0		
	No	749	667	82		

**Notes**: MCI, mild cognitive impairment; CSI-D, Community Screening Instrument for Dementia; AD8, Dementia Screening Questionnaire; PHQ-9, Patient Health Questionnaire Depression Scale; AIS, Athens Insomnia Scale.

### Multiple Logistic Regression Analysis of MCI

Multiple logistic regression was performed using MCI prevalence as the dependent variable and gender (male = 0, female = 1), age, occupation (retired = 0, employed/farming = 1), education level (primary school and below = 0, junior high school and above = 1), and scores on the CSI-D, AD8, PHQ-9, and AIS scales as independent variables. The analysis identified female (*p *= 0.026), high age (*p *= 0.009), low CSI-D score (*p *= 0.007), high AD8 score (*p* < 0.001), and high PHQ-9 score (*p *= 0.037) as significant risk factors for MCI in the elderly urban population of Huzhou City (Table 2).

**Table 2.** **Multiple logistic regression analysis of MCI**.

Variables	B	Standard error	Wald	*p*-value	Odds ratio	95% CI	
						Lower	Upper
Gender	0.671	0.301	4.966	0.026	1.956	1.084	3.530
Age	0.060	0.023	6.808	0.009	1.062	1.015	1.111
Occupation	–0.481	0.340	2.003	0.157	0.618	0.317	1.204
Education level	–0.223	0.318	0.493	0.483	0.800	0.429	1.492
CSI-D	–0.278	0.103	7.257	0.007	0.758	0.619	0.927
AD8	0.451	0.056	64.491	<0.001	1.569	1.406	1.752
PHQ-9	0.140	0.067	4.361	0.037	1.150	1.009	1.312
AIS	–0.066	0.055	1.438	0.230	0.936	0.841	1.043
Constant	–5.365	2.076	6.675	0.010	0.005		

**Notes**: CSI-D, Community Screening Instrument for Dementia; AD8, Dementia Screening Questionnaire; PHQ-9, Patient Health Questionnaire Depression Scale; AIS, Athens Insomnia Scale; CI, confidence interval.

## Discussion

As a transitional state between normal aging and dementia, the prevalence of MCI increases year by year with the intensification of population aging, posing a serious threat to the physical and mental health of the elderly [24]. Studies have shown that early cognitive training, lifestyle modifications, and enhanced social support can effectively stabilize or improve cognitive function in MCI patients, thereby enhancing their quality of life [25, 26]. However, the clinical manifestations of MCI are varied, and some patients may remain stable for a period of time, while others may rapidly progress to dementia. Additionally, the early diagnosis of MCI is challenging due to the lack of specific biomarkers [27]. Therefore, it is of great significance to conduct an in-depth epidemiological investigation of MCI patients.

In this study, 778 elderly people in Huzhou City included 668 people with normal cognitive function, 82 people with MCI and 28 people with dementia. The prevalence rate of MCI was 10.54%. Hu Y *et al*. [28] conducted MCI screening on 5715 people over 60 years old in China and found that the incidence of MCI in the non-sarcopenia group was 10.1%, which was similar to the results in this paper. Jia L *et al*. [29] investigated 46,011 people aged 60 or above from 96 sites in China and found that the total prevalence of MCI was 15.5%. Cong L *et al*. [30] investigated 5068 Chinese residents aged 60 and above, and found that the prevalence rate of MCI was 26.48%. The prevalence rate of MCI in the above studies was significantly higher than that in this survey. Jia L *et al*. [29] surveyed all residents, and Cong L *et al*. [30] surveyed rural residents.

Through further analysis, this study determined that female, high age, low CSI-D score, high AD8 score, and high PHQ-9 score were all risk factors for MCI in the elderly population in Huzhou City.

The risk of MCI in elderly female residents in this region is higher than that of male residents, and the reasons may include the following: differences in physiological structure, estrogen regulation of cognitive-related brain neurons and thinking processes, the risk of MCI in elderly women may increase due to the decrease of estrogen with age. In the early period of China, there was a serious traditional social concept of “son preference”. In addition, due to the low social and economic level and limited national educational resources at that time, women’s education level was generally low, which affected women’s cognitive reserve. In addition, the subjects of this survey are aged 60 and above, most of whom were born in the early years of the founding of the People’s Republic of China, when women’s social participation was lower than that of men.

Lee S *et al*. [31] also proposed that women’s cognitive function was affected by social activities and the number of friends. Chen HF *et al*. [32] and Li W *et al*. [33] respectively conducted cross-sectional studies on patients with type 2 diabetes and schizophrenia, and found that women had a higher proportion of cognitive dysfunction and were inferior to men in terms of overall cognitive function and executive function. Son YJ *et al*. [34] investigated 152 patients in intensive care units in South Korea using a prospective cohort study, and found that the proportion of persistent cognitive impairment in female patients was significantly higher than that in male patients. Yang H *et al*. [35] pointed out that living with others in men with poor sleep quality is conducive to reducing the risk of MCI, but there is no such protective effect in women, and speculated that this may be another factor leading to the higher prevalence of MCI in women. Okamoto S *et al*. [36] also came to a conclusion consistent with this study by taking elderly people in Japan as the investigation objects.

Age has been identified as an independent factor influencing MCI in the elderly population of Huzhou city. This correlation is attributed to age-related memory decline, reduced physical function, and decreased social activity. As people age, there is a gradual reduction in cerebral blood flow, oxygen and glucose metabolism, and blood-brain barrier function. Concurrently, brain tissue undergoes organic changes and atrophy, leading to cognitive dysfunction across multiple domains. Additionally, after retirement, the elderly often experience reduced mental engagement, decreased participation in social activities, and diminished exposure to new information, all of which contribute to cognitive decline. The CSI-D and AD8 scales effectively detect early-stage dementia, and their scores correlate directly with MCI, reinforcing their utility in assessing cognitive impairment.

This study also found that a high PHQ-9 score is a significant risk factor for MCI, indicating a strong association between depressive symptoms and MCI in the elderly. Numerous studies have explored the biological mechanisms underlying cognitive decline due to depression [37, 38, 39, 40]. Some suggest that depression disrupts the neuroendocrine axis, increasing corticosteroid secretion and leading to hippocampal neuron death and neural inhibition (endocrine hypothesis). Others propose that depressive symptoms cause glial cell damage, trigger inflammatory factors, and lead to myelin degradation (neuroinflammatory hypothesis). Additional theories include the vascular depression hypothesis, neurotrophic factor hypothesis, and amyloid hypothesis. Hou Z *et al*. [41] found that the intensification of depressive symptoms was significantly related to the rapid decline of cognitive ability. Shin M [42] investigated elderly people in South Korea and found that depressive symptoms could increase the risk of cognitive dysfunction. Ma W *et al*. [43] took 7525 Chinese elderly people as investigation objects and found that depression, social relations and cognitive function were significantly correlated. Alexopoulos P *et al*. [44] also identified a link between cognitive dysfunction and depressive symptoms in patients with rheumatoid arthritis (RA) and systemic sclerosis. These findings highlight the need for early intervention to address depressive symptoms and mitigate their impact on cognitive function in elderly populations.

This study has several limitations: (1) The sample was limited to eight residential communities in two streets of Wuxing District, Huzhou, which may not fully represent the elderly population aged 60 and above in the area; (2) The CSI-D, AD8, PHQ-9, and AIS questionnaires used may be influenced by subjective factors; (3) This is a cross-sectional study, and MCI is a progressive condition, meaning that new cases may continue to emerge; (4) Other studies have linked MCI to factors such as hearing impairment, enteritis, and thyroid function, which were not observed in this study, possibly due to limited sample size. In the follow-up study, we will expand the sample size, survey scope and survey time, and comprehensively consider diet, exercise and other lifestyle factors to confirm the universal significance of the findings.

## Conclusion

This study provides valuable insights into the prevalence and influencing factors of MCI among the elderly population in Huzhou City, revealing a prevalence rate of 10.54%. MCI incidence was found to be significantly associated with gender, age, health status, and depressive symptoms. Given these findings, it is necessary to focus on these high-risk groups and implement targeted interventions as early as possible to enhance cognitive function and quality of life among the elderly population.

## Availability of Data and Materials

The datasets used and/or analyzed during the current study are available from the corresponding author on reasonable request.
